# A Case of Neurosarcoidosis with Labyrinthine Involvement

**DOI:** 10.1155/2014/530431

**Published:** 2014-03-06

**Authors:** Peter B. Johnson, Roxanne Melbourne-Chambers, Amit Manohar Saindane, Nilesh Desai, Myrton Smith

**Affiliations:** ^1^Department of Surgery, Radiology, Anaesthetics and Intensive Care, Faculty of Medical Sciences, University of the West Indies (Mona Campus), Kingston, Jamaica; ^2^Department of Child Health, Faculty of Medical Sciences, University of the West Indies (Mona Campus), Kingston, Jamaica; ^3^Department of Radiology and Imaging Sciences, School of Medicine, Emory University, 100 Woodruff Circle, Atlanta, GA 30322, USA

## Abstract

Sarcoidosis is a chronic granulomatous disease of unknown aetiology, which may involve any organ system. It most commonly occurs in adults with childhood involvement being rare. Central nervous system involvement is seen in up to 25% and typically involves meningeal disease resulting in multiple cranial neuropathies. Other common clinical findings include seizures, headache, dementia, and pituitary dysfunction. Imaging plays a central role in the diagnosis with typical findings including pachymeningeal and leptomeningeal enhancing lesions. Other imaging findings include lacunar and major territory infarcts, hypothalamic and infundibular thickening, hydrocephalus, and cranial nerve enhancement. We present a case of an eight-year-old male patient with progressive headache, visual disturbance, unilateral sensory hearing loss, and multiple cranial neuropathies. Imaging findings demonstrated the classic pachymeningeal and leptomeningeal enhancement along much of the skull base, as well as enhancement of the right and left second and eighth cranial nerves. Extensive inflammatory changes were noted in the temporal bones and paranasal sinuses. There was also enhancement of the right and left labyrinths. Sinus biopsy confirmed sarcoidosis. We present the first case to our knowledge of sarcoid labyrinthitis.

## 1. Introduction

Sarcoidosis is a chronic granulomatous disease of unknown aetiology with the presence of noncaseating granulomas [1]. It may involve any organ system, although the lungs and lymphatic system are among the more common sites. Sarcoidosis typically affects adults, with bimodal peaks in the third decade and sixth decade [1]. It is uncommon in childhood. There is an increased risk in people of African ethnicity [2], although this seems to be more prevalent in those living in northern climates. Isolated neurosarcoidosis is rare [3]. In postmortem series of patients with sarcoidosis, central nervous system involvement is seen in 14–25% [4–7]. Symptomatic cases are however reported with a much lower frequency (3%–5%) [8, 9]. This suggests that CNS involvement is more common than clinically apparent.

Depending on the organ systems involved, clinical findings may vary widely. With central nervous system involvement, symptoms include those of cranial neuropathy, particularly the facial nerve and the optic nerve. Other symptoms include seizures, headache, dementia, weakness, parasthesia, and pituitary/hypothalamic dysfunction [10].

## 2. Case Report

We present a case of an eight-year-old male with a progressive history of headache, neck pain, and deteriorating vision over a six-month period. Approximately one month prior to presentation, he developed right facial weakness and unilateral tongue weakness. There was no history of seizures, fever, gastrointestinal symptoms, respiratory symptoms, or exposure to heavy metals. His immunization record was current and he had no recent vaccinations. No changes in behavior or cognition were reported. Clinical examination revealed blindness in the right eye and impaired vision in the left eye. On formal ophthalmologic examination, right optic disc pallor was present with an afferent pupillary defect. Bilateral oculomotor and abducens nerve palsy were present. Lower motor palsy of the right and left facial nerves as well as right accessory and right hypoglossal nerve was noted. There was hearing loss of the right ear and Weber test lateralized to the left ear. There was, additionally, mild weakness of the soft palate suggesting involvement of the glossopharyngeal and vagus nerves. Right hemiparesis and motor weakness of the left lower limb were noted. The growth parameters were normal.

Laboratory investigations revealed a hypochromic, microcytic anemia; marked elevation of the sedimentation rate; cerebrospinal fluid pleocytosis; hyperglobulinemia; normal serum calcium; and alkaline phosphatase. The tuberculin skin test was negative and microscopy and staining of gastric washings for acid fast bacilli were negative.

**Figure 1:**
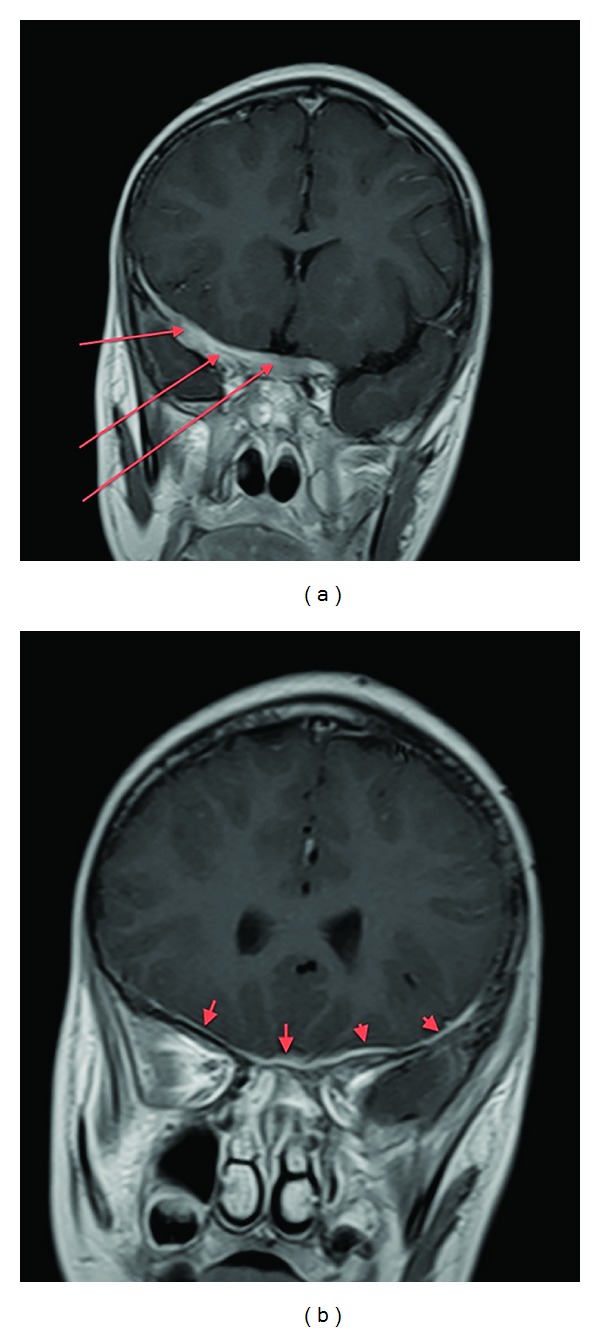
Coronal post-Gad T1WI FSE. There is diffuse pachymeningeal enhancement along the anterior skull base (red arrows).

**Figure 2:**
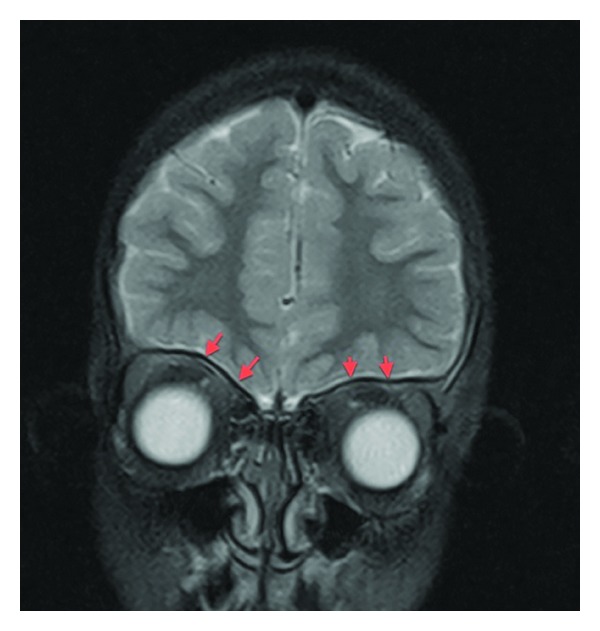
Coronal T2WI FSE. There is diffuse hypointense dural thickening (red arrows).

**Figure 3:**
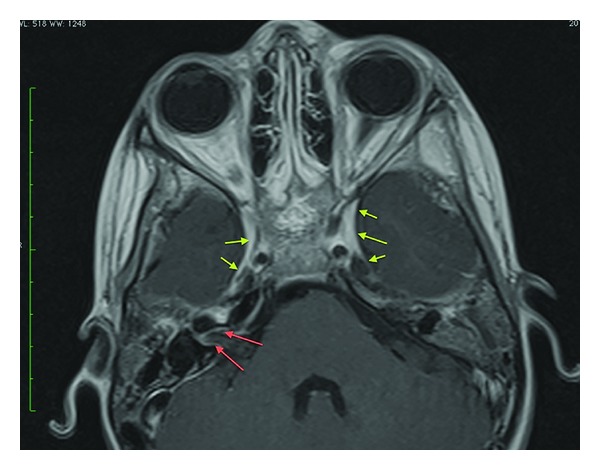
Axial post-Gad T1WI FSE. Dural enhancement is noted along the cavernous sinuses (yellow arrows). There is dural enhancement extending into the right internal auditory canal, with abnormal enhancement of the facial and vestibulocochlear nerves (red arrows).

**Figure 4:**
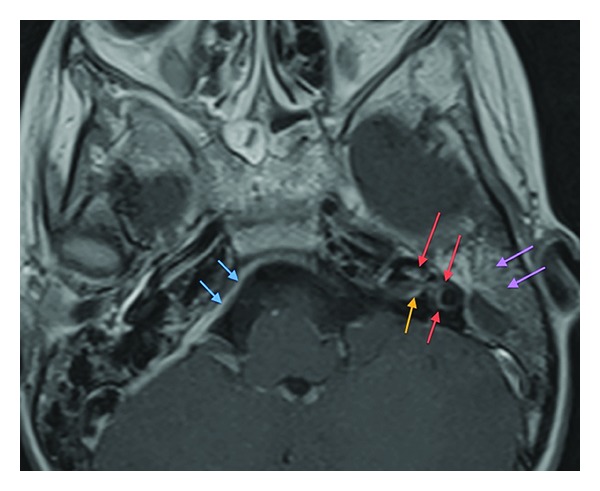
Axial post-Gad T1WI FSE. Thick dural enhancement is noted in the posterior fossa (blue arrows). There is enhancement in the left internal auditory canal (orange arrow). There is left mastoid sinus opacification with enhancement (purple arrows). There is enhancement of the entire right labyrinth (red arrows).

**Figure 5:**
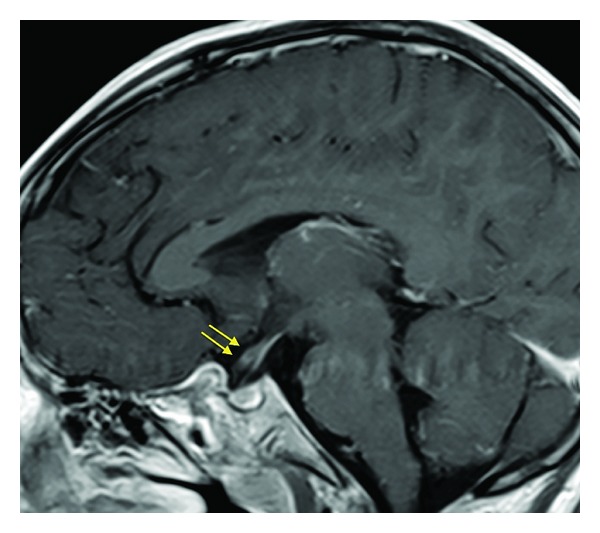
Sagittal post-Gad T1WI FSE. Enhancement of the right optic nerve (yellow arrows).

At presentation to our institution, the patient had undergone CT and MRI of the brain; the former was not available for formal review but was reported as normal. MRI revealed extensive smooth pachymeningeal thickening and enhancement involving the anterior, middle, and posterior skull base. This included thick pachymeningeal enhancement along the lateral dural margin of the right and left cavernous sinuses, the right and left petroclinoid ligaments, and along the right petrous temporal bone inferiorly to the level of the foramen magnum (Figures 1, 3, and 4). These areas of dural enhancement and thickening were isointense on noncontrast T1 FSE and hypointense on T2 FSE (Figure 2). There was also dural enhancement of the walls of the right and left internal auditory canals (more extensive on the right) as well as the cisternal and intracanalicular segments of the seventh and eighth cranial nerves (Figures 3 and 4). There was no apparent involvement of the petrous, tympanic, or descending segments of the facial nerves. Mild enhancement of the leptomeningeal surfaces of the intracranial portion of the right optic nerve, the optic chiasm, and the bilateral optic tracts was noted (Figure 5).

Mild leptomeningeal enhancement was also noted along the basal surfaces of the brain.

**Figure 6:**
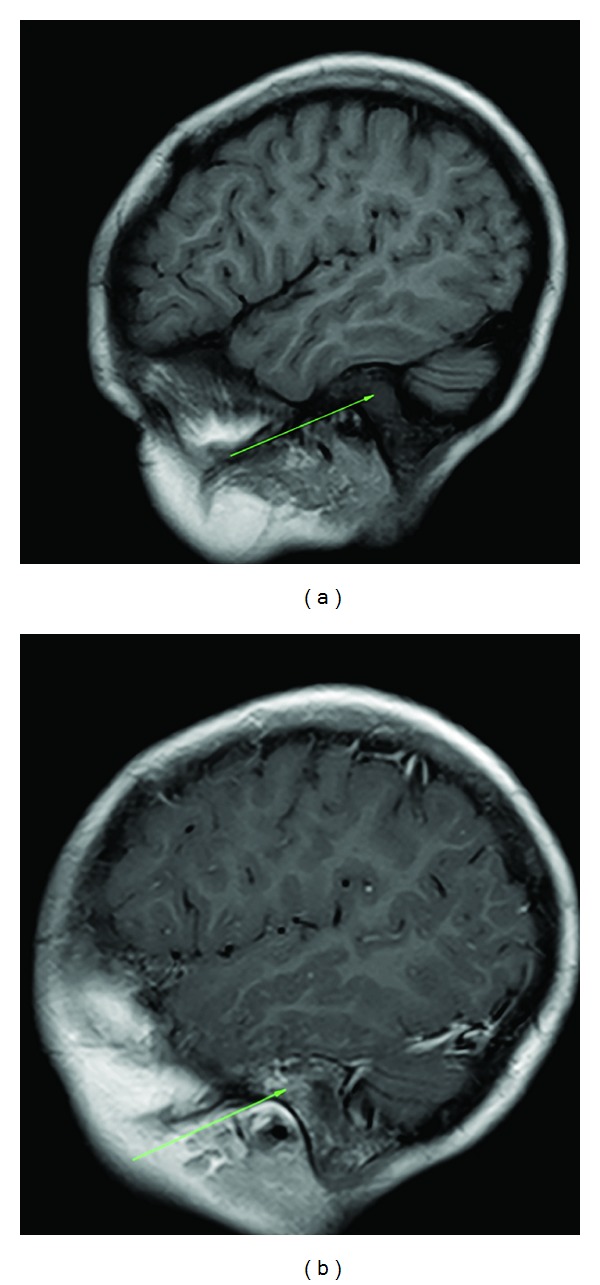
(a) Precontrast T1 FSE. Opacified left mastoid sinus. (b) Postcontrast T1 FSE. There is extensive mural enhancement postcontrast.

Interestingly, there was enhancement of the cochlea, semicircular canals, and vestibule on the left (Figure 4). There was also fluid opacification of the left mastoid sinus air cells and tympanic cavity with extensive mural enhancement (Figures 4 and 6). Similar but less extensive changes were also noted in the right mastoid sinus and tympanic cavity. Subsequent CT of the temporal bones demonstrated similar changes in the mastoid sinuses with no associated bony destruction.

**Figure 7:**
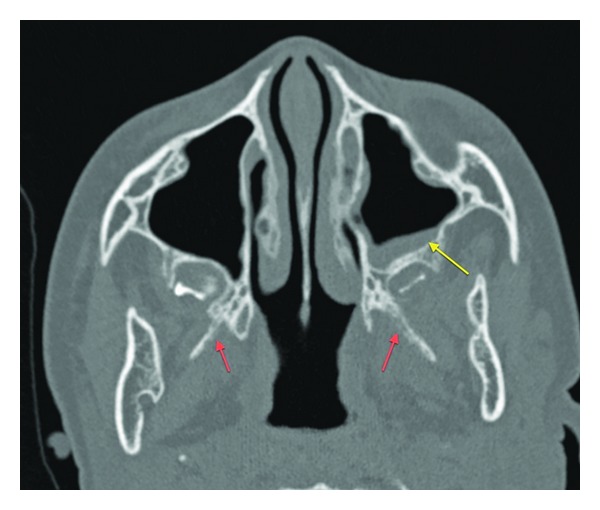
Axial CT: mucosal thickening of the left maxillary antrum is noted with mural bony thickening (yellow arrow). There are erosive changes involving the lateral pterygoid plates (red arrows).

Subtle erosive changes were also noted involving the right and left medial and lateral pterygoid plates. There was mild generalized mucosal thickening involving the right and left maxillary sinuses and the anterior and posterior ethmoidal sinuses with thickening of the associated bony walls (Figure 7).

The patient had an otolaryngology consult and a biopsy done of the floor and medial wall of the right maxillary sinus and wall of the right ethmoid sinus. The final pathology report of that biopsy revealed sarcoidosis.

## 3. Discussion

Imaging findings in neurosarcoidosis are quite varied and include varying patterns of pachymeningeal and leptomeningeal enhancement, dural based masses, ischaemic intra-axial lesions (lacunar and major territory infarcts), hypothalamic and infundibular thickening, hydrocephalus, and cranial nerve enhancement [10]. Many of these findings may be seen intracranially and within the spine. The most commonly found finding however is T2 hypointense dural thickening and enhancement [10–12]. This is thought to be secondary to fibrocollagenous tissue associated with sarcoid. While a common feature of neurosarcoidosis, such an appearance of pachymeningeal involvement, may be seen with entities such as Wegener granulomatosis, idiopathic hypertrophic cranial pachymeningitis, and rheumatoid nodulosis [10]. When sarcoidosis involves the dura more focally, it may be confused with meningiomas and dural metastases. Cranial nerve involvement is reported to be present at MRI in less than 50% of cases [3, 10, 13]. The inflammatory process may spread along the perivascular spaces resulting in vasculitis. This results in varying degrees of ischaemic changes. At MRI these range in appearances from lacunar infarcts to larger territory infarction. The MRI findings may not correlate with the clinical symptoms [10, 11, 14]. Spinal cord involvement is seen in approximately one-quarter of patients and typically appears as fusiform enlargement of the cervical and thoracic cord with associated increased T2 signal intensity. Its appearance therefore may be confused with multiple sclerosis and other demyelinating lesions of the spinal cord as well as infectious myelitis.

While involvement of the eighth cranial nerve is well described in the literature, both in terms of clinical and imaging findings [15], we have not found any report of labyrinthine involvement in sarcoidosis. In our case, there was enhancement of the labyrinths indicating labyrinthitis along with coexisting otomastoiditis. There are several possible mechanisms of involvement of the labyrinth including direct spread from the inflamed temporal bone structures as well as venous spread. Our case represents the second case of neurosarcoidosis in a paediatric patient at the University Hospital of the West Indies within the last decade. It also represents, to our knowledge, the first reported case of labyrinthine involvement by sarcoidosis diagnosed at MRI.
